# Electronic Properties of Vanadium Atoms Adsorption on Clean and Graphene-Covered Cu(111) Surface

**DOI:** 10.1186/s11671-018-2605-3

**Published:** 2018-07-06

**Authors:** Yi-Xu Xu, Xin-Rui Cao, Lin-Han Xu, Jian-Hua Zhang, Shun-Qing Wu, Zi-Zhong Zhu

**Affiliations:** 1 Collaborative Innovation Center for Optoelectronic Semiconductors and Efficient Devices, Department of Physics, Jiujiang Research Institute, Xiamen University, Xiamen, 361005 China; 2 Fujian Provincial Key Laboratory of Theoretical and Computational Chemistry, Xiamen University, Xiamen, 361005 China; 3 Institution of Electromagnetics and Acoustics, and department of Electronic Science of Xiamen University, Xiamen, 361005 China

**Keywords:** Vanadium, Adsorption, Graphene, Cu(111) surface, First-principles calculations

## Abstract

The electronic properties of vanadium atoms adsorbed on clean and graphene-covered Cu(111) surface have been systematically studied using ab initio theoretical method. Two coverages (1/9 ML and 1 ML) of vanadium adsorption are considered in this work. Our calculations indicate that V staying underneath the Cu surface is found to be the most stable adsorption site at the aforementioned two coverages for V/Cu(111). However, such adsorption may lead to undesired properties. Therefore, we introduce graphene as a buffer layer to effectively alleviate the direct interaction between V and Cu surface. The calculations show that electronic properties of the original graphene layer are significantly affected by the interactions of C atoms with the V adatoms; the Dirac point of graphene is “destroyed” as a consequence at both coverages. In the V/Gra/Cu(111) system, the interaction between graphene layer and the substrate Cu atoms remains weak as in the Gra/Cu(111) system. Moreover, a relatively low coverage of 1/9 ML gives rise to a spin-polarized system while a non-spin-polarized system is observed at the coverage of 1 ML. This finding offers a new way for the application of vanadium-based materials in reality.

## Background

Heterogeneous catalysis plays a crucial role in many areas of chemical and energy industries. Intensive studies have focused on understanding, improving, and designing new catalysts by now. Adsorption of transition metal atoms on noble metal substrate can influence the corresponding catalytic properties, which is one of the most significant topics in catalysis [1–7]. In particular, the adsorption of one monolayer metal on metal surface exhibits significantly different chemical and catalytic properties within various kinds of adsorbed systems [5–7]. In general, the catalytic properties of the materials depend on their atomic structures, composition, and the electronic states that near the Fermi level [8–12]. The substrate is expected to directly and/or indirectly influence the catalytic properties of metal deposits. As we all know, Cu(111) surface is one of the most thoroughly investigated single crystal metal surfaces in the past few decades [13–24]. Especially, in the last decade, Cu(111) surface has been considered as the most primary substrate for the growth of high-quality and large-area graphene by chemical vapor deposition (CVD) [22–24]. The novel electronic properties of graphene can be well preserved on such a substrate. The adsorption of late 4d transition metals (such as Rh [25], Pd [26–30], Ir [31], and Pt [29, 32, 33]) on the Cu(111) surface have been widely studied both experimentally and theoretically. However, the study of the early 3d transition metal atoms adsorbed on the Cu(111) surface is relatively lacking [34–37]. Here, we focus on the early 3d transition metal element, vanadium, due to its biochemical relevance and extensive applications in several industrial fields, such as heterogeneous catalysis, molecular networks, nanomaterials, and building of batteries [38]. The vanadium-based polyanion materials are proposed to be the candidates to replace the commercial cathode materials LiCoO_2_ and LiMn_2_O_4_ due to its flexible valence states [39]. Hence, studying the adsorption characteristics of vanadium atoms can facilitate its applications in reality. The possible applications of the systems studied can be expected as below. (1) The common oxidation states of the vanadium can be + 2, + 3, + 4, and + 5; therefore, it can be used as a powerful and versatile catalyst in nanomaterials industry [38]. (2) Vanadium in the metallic state can be used to catalyze the disproportionation of CO to C and CO_2_ [40]. (3) It is also interesting to analyze the TM (i.e., vanadium) atoms adsorbed on surfaces with a weak concentration of free electrons due to the possible increasing of the electrical and thermal conduction [41]. Moreover, there is an extraordinary interest in the magnetic order in two-dimensional surface systems that can be used in recording media, magnetic inks, and spintronic devices.

In this work, we report a systematic investigation of the adsorption of vanadium atoms on clean Cu(111) surface and on graphene-covered Cu(111) surface based on the density functional theory (DFT). For the abovementioned two systems, two contrastive coverages (i.e., 1/9 ML and 1 ML) of the vanadium adatoms are considered to evaluate the effect of coverage on the electronic and magnetic properties. The lowest energy adsorption site for V adsorbed on the clean Cu(111) surface is underneath the surface rather than above the surface regardless of the V coverages. For the adsorption of V on the graphene-covered Cu(111) surface, the adsorption sites are coverage-dependent, that is, the hollow site with maximum coordination is energetically favored for 1/9 ML coverage, while the top site with low coordination is preferred for 1 ML coverage. Meanwhile, for both V/Cu(111) and V/Gra/Cu(111) systems, the spin polarizations of V adatoms are energetically favored for the 1/9 ML coverage while no magnetism is found for the 1 ML coverage. Additionally, a net magnetic moment for C atoms of graphene is about 0.16 μ_B_/per carbon for 1/9 ML V/Gra/Cu(111) system, which is different from the results in the system of Gra/Cu(111). To gain a deep understanding of the interactions in the V/Cu(111) and V/graphene/Cu(111) systems, the electronic states at the Fermi surface are analyzed in detail. In brief, our studies could help understanding the electronic properties of V/Cu(111) and V/Gra/Cu(111) systems.

## Methods

Our calculations have been performed by employing the Vienna ab initio simulation package (VASP) [42] which is based on the spin-polarized density functional theory [43], the plane wave basis, and the projector augmented wave (PAW) representation [44]. The Perdew-Burke-Ernzerhof (PBE) exchange-correlation energy functional [45] within the generalized gradient approximation (GGA) is employed in the calculations (some comparative studies using B3LYP [46, 47] and HSE06 [48] hybrid functionals are also presented when necessary). In order to accurately describe the van der Waals (vdWs) interactions between graphene and Cu(111) surface, the PBE functional with the vdWs correction (DFT-D2) [49] is adopted. The cutoff plane-wave kinetic energy is set to be 500 eV. The Cu(111) surface was modeled by using a slab model that comprises seven Cu layers together with a vacuum spacing of about 20 Å. Different V coverages on Cu(111) and Gra/Cu(111) surfaces were modeled by using different supercells. For 1/9 ML and 1 ML of V coverages, we employed (3 × 3) and (1 × 1) surface unit cells, respectively. The Monkhorst-Pack scheme [50] with a 24 × 24 × 1 k**-**mesh was used to sample the Brillouin zone integration for the (1 × 1) surface unit cell, while an 8 × 8 × 1 k**-**mesh was used for the (3 × 3) surface unit cell. During the optimization, the lowermost three Cu layers of the slab were frozen while the remaining atoms of the systems were fully relaxed until the force on each atom was less than 0.01 eV/Å. Vanadium atoms were adsorbed on one side of the slab. The dipole correction [51] is not considered in this study due to a negligible energy correction found on the basis of our calculations.

## Results and Discussion

### Vanadium Atom Adsorption on Clean Cu(111) Surface

**Fig. 1:**
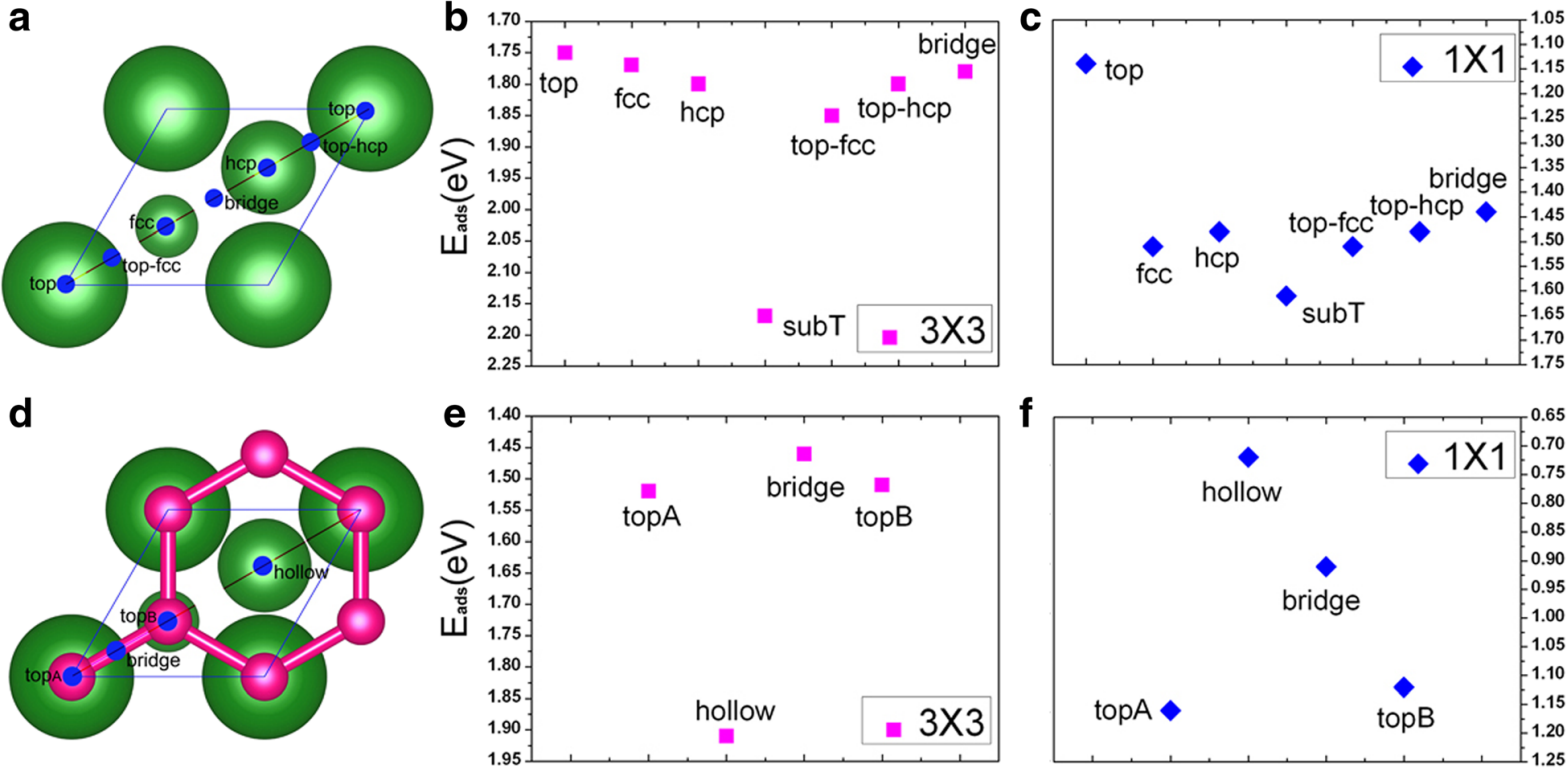
**a** Adsorption sites on a clean Cu(111) (1 × 1) surface: largest balls indicate surface Cu atoms, and smaller balls indicate sub-layer Cu atoms; **b**, **c** adsorption energies of different adsorption sites of V atoms on Cu(111) at 1/9 ML and 1 ML coverages, respectively; **d** a graphene-covered Cu(111) (1 × 1) surface, red balls indicate C atoms of graphene; **e, f** adsorption energies of V atoms on graphene-covered Cu(111) at 1/9 ML and 1 ML coverages, respectively

**Fig. 2:**
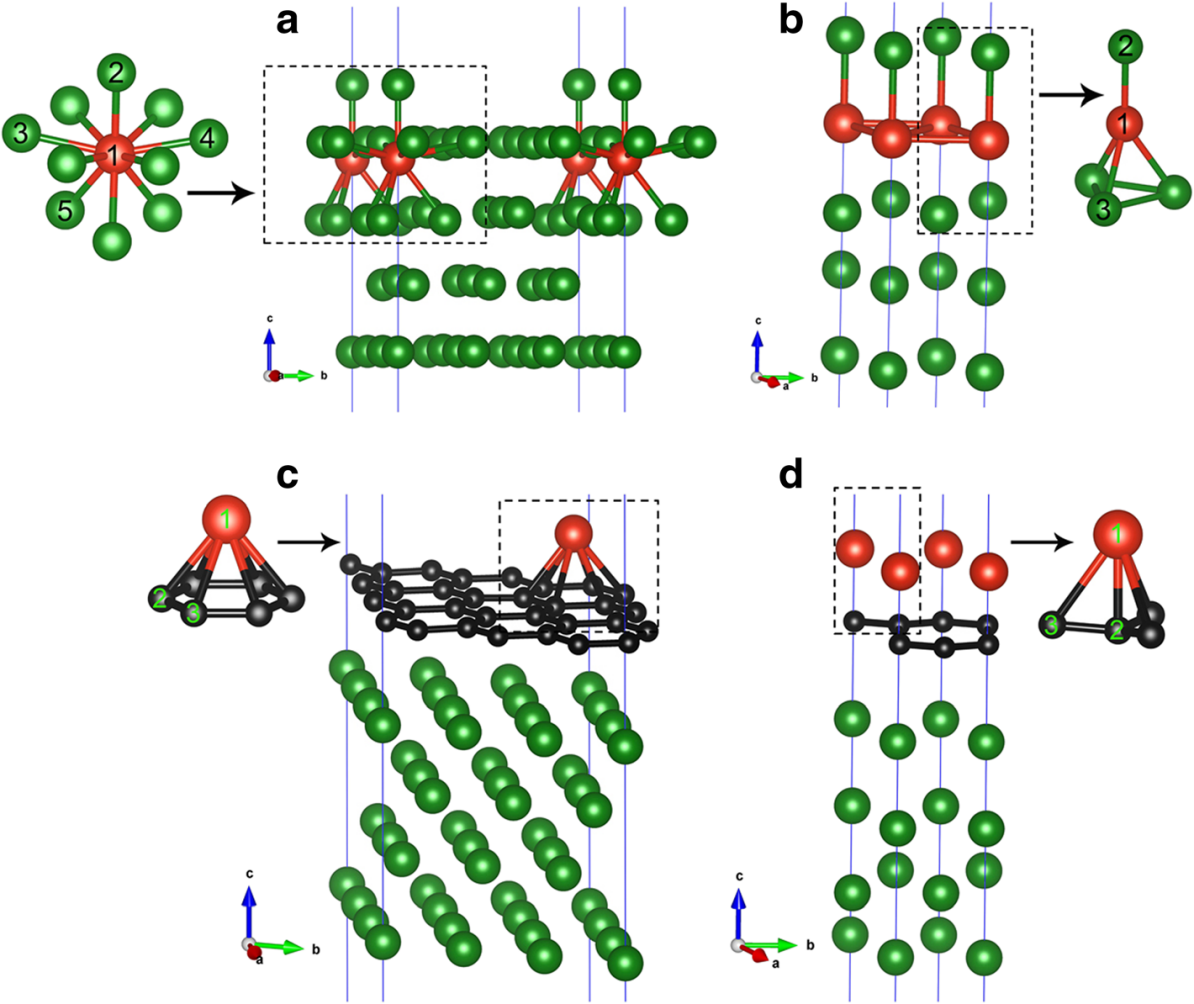
Geometries of V/Cu(111) systems for **a** 1/9 ML and **b** 1 ML coverages. Geometries of V/Gra/Cu(111) systems for **c** 1/9 ML and **d** 1 ML coverage of V atoms. Red, black, and green balls represent V, C, and Cu atoms, respectively

In this section, we present results for the V adsorption directly on the clean Cu(111) surface at two coverages (i.e., 1/9 ML and 1 ML). To find a favorable adsorption site of V atom on the Cu(111) surface, seven possible adsorption sites are considered for each coverage, that is, the top, fcc, hcp, subT, top-fcc, top-hcp, and bridge site, as shown in Fig. 1a. In particular, it should be noted that the subT is a site underneath the Cu surface, where V adatom exchanges its position with the surface layer Cu atom (and moves Cu atom to the site directly above the vanadium atom); see Fig. 2a, b. For both adsorption coverages, the adsorption energies for all the seven adsorption sites are calculated for the V/Cu(111) system. The obtained results are given in Fig. 1b, c. Here, the adsorption energy per vanadium atom (E_ad_) is calculated by the following formula:

$$ {\mathrm{E}}_{\mathrm{ad}}=\left[\left({NE}_V+{E}_{Cu(111)}\right)-{E}_{V/ Cu(111)}\right]/N $$

where *E*_*V*_ is the energy of an isolated vanadium atom, *E*_*Cu*(111)_is the total energy of a clean Cu(111) surface involved, *E*_*V*/*Cu*(111)_ is the total energy of a V/Cu(111) system, and *N* is the number of V atoms involved. From Fig. 1b, c, one can see that the subT sites are energetically favored for the V adsorptions on Cu(111) surface for the aforementioned coverages. Based on it, we will consider only the subT sites in the following discussions. The calculated adsorption energies, the bond lengths between V atom and its adjacent Cu atoms, and the atomic magnetic moments of V adatoms for the V/Cu(111) are listed in Table 1. As we can see from Table 1, the adsorption energies E_ad_ are 2.17 and 1.61 eV per V atom for 1/9 ML and 1 ML coverages, respectively, which indicates that the interaction of V atoms with the Cu(111) surface is quite strong. Moreover, the adsorption energy reduces with the increase of V coverage, which means that V-V interactions become stronger while the interactions between the V layer and the Cu surface become weaker. The shortest bond lengths between the V atom and its adjacent Cu atoms are 2.27 and 2.37 Å for 1/9 ML and 1 ML coverages, respectively. This implies that the interaction between the V adatom and the Cu substrate is relatively stronger for 1/9 ML, which is in consistent with the calculated results from adsorption energy. The ferromagnetic (FM) order of V adatoms is also considered in the calculations, and the spin polarization energy of the FM order is calculated by Δ*E* = (*E*_*no*_*mag*_ − *E*_*FM*_)/*N* (with *E*_*no*_*mag*_ is the energy of non-magnetic state). The spin polarization energy of a vanadium atom is 110 meV for the 1/9 ML coverage (see Table 1), while there is no magnetism for the 1 ML coverage. The atomic magnetic moment of V is 1.34 μ_B_ for the 1/9 ML coverage of vanadium, which is very much different from the value (3 μ_B_) of a gas-phase V atom. We will discuss on this point later.

**Table 1:** Adsorption energies at subT sites E_ad_, spin polarization energies (per V atom) of V atoms ∆E, the bond lengths between V atom and its adjacent Cu and C atoms *d*_V-Cu_ and *d*_V-C_, the vertical distance between the lower C layer and the uppermost Cu layer *d*_(Gr-Cu)⊥_, and atomic magnetic moments of V and C atoms *M*_V_ and *M*_C_. For the subscript of Cu and C atoms, please refer to Fig. 2

V/Cu(111) coverage (ML)	*E*_ad_ (eV)	∆*E* (meV)	*d*_V-Cu2_ (Ǻ)	d_V-Cu3_ (Ǻ)	*d*_V-Cu5_ (Ǻ)	*M*_V_ (μB)	
1/9	2.17	110	2.27	2.51	2.40	1.34	
1	1.61	0	2.37	2.63	–	0.00	
V/Cu(111) coverage (ML)	*E*_ad_ (eV)	∆*E* (meV)	*d*_V-Cu2_ (Ǻ)	*d*_V-Cu3_ (Ǻ)	*d*_(Gr-Cu)┴_ (Ǻ)	*M*_V_ (μ_B_)	*M*_C_ (μ_B_)
1/9	1.91	390	2.31	2.31	3.04	2.93	0.16
1	1.16	0	2.12	2.46	2.83	0.00	0.00

**Fig. 3:**
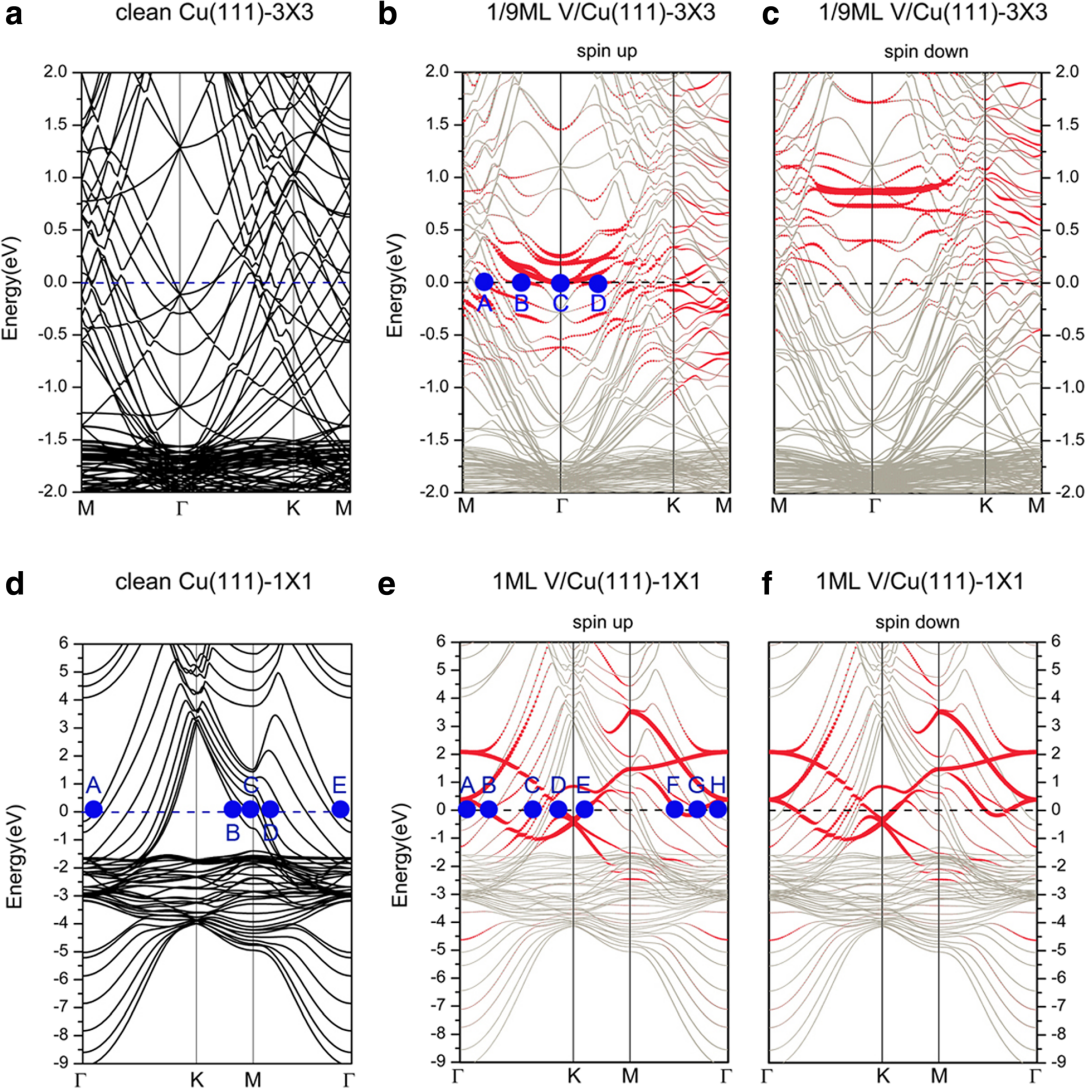
Band structures of a clean Cu(111) surface, plotted in the Brillouin zones (BZ) of **a** 3 × 3 unit cell and **d** 1 × 1 unit cell. Band structures of V adsorption on Cu(111) surface with 1/9 ML coverage for **b** spin up and **c** spin down. Band structures of V adsorption on Cu(111) surface with 1 ML coverage for **e** spin up and **f** spin down

Next, we discuss the electronic structures of the V/Cu(111) systems. The band structures of V adsorptions on Cu(111) (3 × 3) and Cu(111) (1 × 1) surfaces (i.e., 1/9 and 1 ML) are presented in Fig. 3, and the band structures of the corresponding clean Cu(111) (3 × 3) and Cu(111) (1 × 1) surfaces are also plotted for comparison. Both Fig. 3a, d can be used to discuss on the electronic structures of a clean Cu(111); we choose here Fig. 3d. We found that both *s* electrons and *d* electrons of Cu contribute to the conductance of the system for the clean Cu(111) surface. In more detail, we labeled the representative points (A, B, C, D, E) on the Fermi surface in Fig. 3d. Points A and E are mainly contributed from the *d*_*yz*_ electrons of the surface Cu atoms. Points B and C show the contributions from the *d*_*xy*_ and *d*_*x*_^*2*^ _*− y*_^*2*^ electrons of Cu atoms, respectively. Point D describes the mixing of *s* electrons with *d*_*z*_^*2*^ and *d*_*x*_^*2*^ _*− y*_^*2*^ electrons between the neighboring Cu atoms. When V is adsorbed on the Cu(111), the obtained band structures change in a different way as the V coverage varies. For 1/9 ML coverage (shown in Fig. 3b, c), the band structures in spin-up and spin-down channels are different, indicating a spin-polarized feature. In Fig. 3, the red points represent the contributions from the V adatoms while the silver gray points show the contributions of the background Cu. From the spin-up channel (i.e., majority spin), both of the *d* electrons of V adatoms and the substrate Cu atoms contribute to the electronic states at Fermi surface significantly. The hybridizations of the *d* electrons of surface Cu atoms and V adatoms are clearly visible. To describe clearly, we also labeled some representative points (A, B, C, D) on the Fermi surface in Fig. 3b. Therein, point A shows the mixing of *d*_*z*_^*2*^ electrons of V adatoms with *d*_*yz*_, *d*_*z*_^*2*^ electrons of the surface Cu atoms. All B, C and D points indicate the contributions from the *d* electrons of V adatoms. As an example, point B demonstrates the contribution from only the *d*_*x*_^*2*^_*-y*_^*2*^ electrons of V adatoms. For the spin-down channel (i.e., minority spin), the band structures show that electronic states that contributed from V adatoms are all well above the Fermi level (unoccupied). The contribution to the Fermi surface is mainly from *s*, *d* electrons of Cu atoms, with quite minor contributions from electrons of V adatoms. The existent difference between these two spin channels indicates a magnetic moment on V adatoms (1.34 μ_B_). For 1 ML coverage (shown in Fig. 3e, f), different from the situation in 1/9 ML, the adsorbed system is non-spin-polarized. For the sake of convenience, we also labeled the representative points (A, B, C, D, E, F, G, H) on the Fermi surface in Fig. 3e. Both the points A and H correspond to the contributions of *d*_*yz*_ electrons of the bottom layers of Cu atoms. The electronic states at points B, D, E, F, and G are contributed by *d* electrons of V adatom. For example, only *d*_*x*_^*2*^ _*− y*_^*2*^ and *d*_*xz*_ of V adatoms contribute to the electronic states at B and D, respectively. For those points between B and D on the Fermi surface, we found a complex mixing of *d* electrons of V adatoms with the *s, d* electrons of the Cu atoms around V atoms. For example, for point C, the electronic structures are characterized by mixing of *s, d*_*yz*_*,* and *d*_*z*_^*2*^ electrons of the surface layer Cu and the topmost sublayer Cu atoms with the d_z_^2^ electrons of V adatoms.

**Fig. 4:**
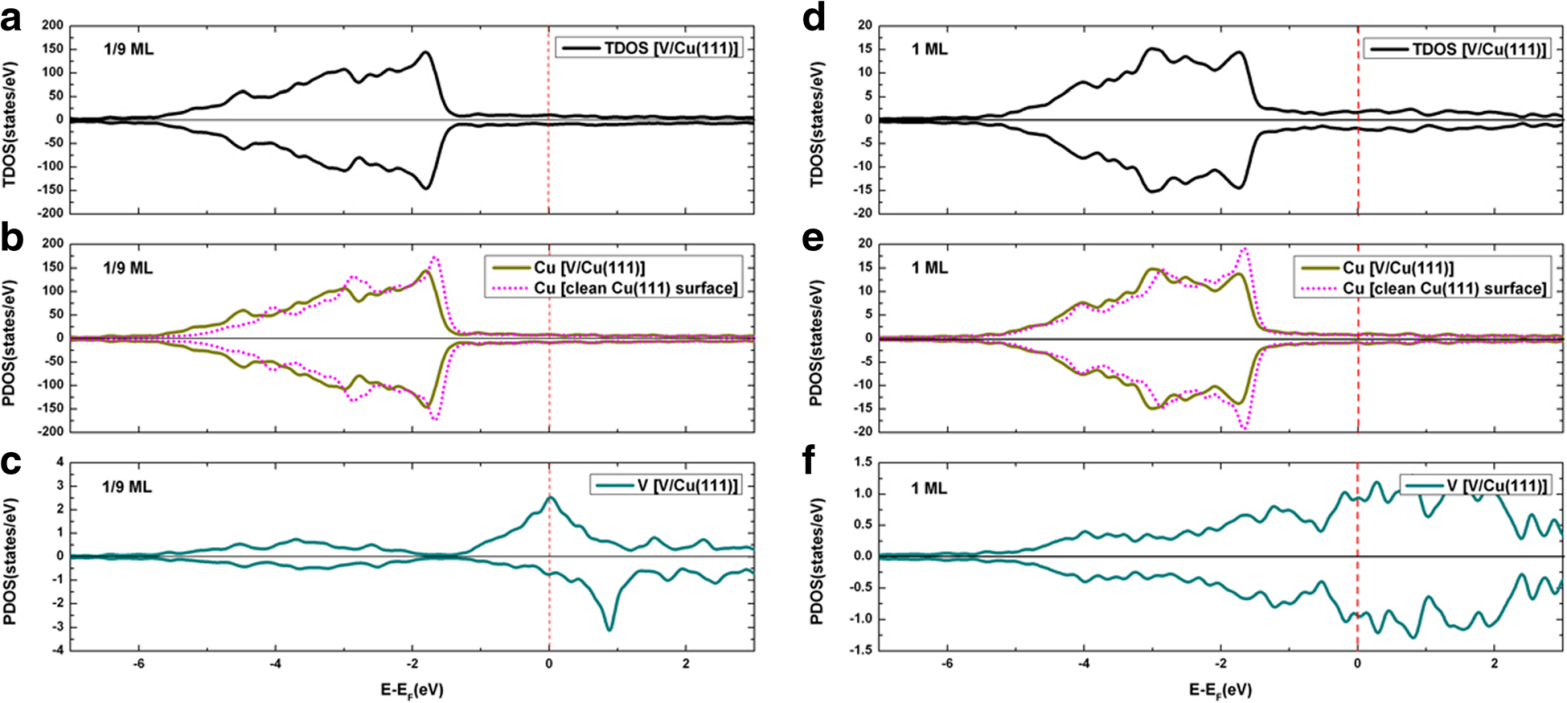
**a** TDOS of V/Cu(111) system at 1/9 ML coverage; **b** PDOS of overall Cu atoms of substrate at 1/9 ML; **c** PDOS for V adatom at 1/9 ML; **d** TDOS of V/Cu(111) system at 1 ML; **e** PDOS for overall Cu atoms of substrate at 1 ML; and **f** PDOS for V adatoms at 1 ML. Notably, the DOS of clean Cu(111) surface is also implanted in graphs **b** and **e** for comparison

**Fig. 5:**
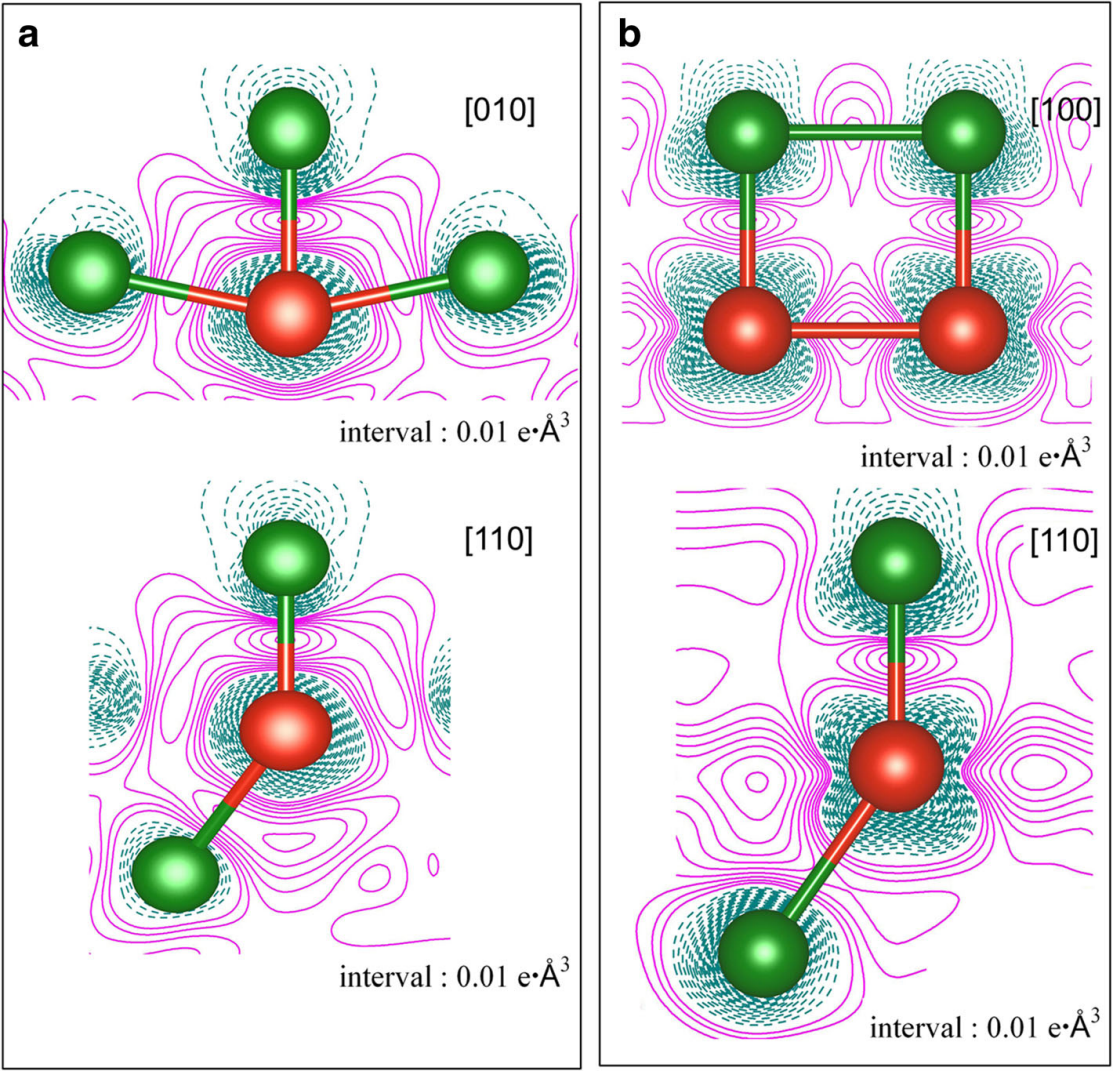
Deformation charge densities for the adsorption of V atoms on Cu(111) surface at two coverages, i.e., **a** 1/9 ML and **b** 1 ML. Electron accumulation and depletion are represented by magenta solid lines and dark cyan dash lines, respectively. The green and red balls represent Cu and V atoms, respectively

The total density of states (TDOS) of V adsorption on the clean Cu(111) surface, together with the projected density of states (PDOS), are demonstrated in Fig. 4 for both the 1/9 ML and 1 ML coverages. Clearly, an obvious spin polarization of V adatoms is found at 1/9 ML (see Fig. 4c) while no spin polarization of V adatoms is found at 1 ML (see Fig. 4f). Moreover, no spin polarization is observed for Cu atoms at both the 1/9 ML and 1 ML coverage (see Fig. 4b, e). By integrating the PDOS of Cu before and after the V adsorption (i.e., leading to the number of electrons on the Cu), we found that the charge on the Cu atoms are slightly increased, indicating a charge transfer from V adatom to Cu substrate in V/Cu(111). In other words, the V adsorption leads to an n-type doping on Cu. To better understand the V adsorption on Cu(111) surface, we plot the contour figures of the deformation charge densities at 1/9 ML and 1 ML coverages in Fig. 5a, b, respectively. The deformation charge densities is defined by $\Delta \rho \left(\overrightarrow{r}\right)={\rho}_{\left[V/ Cu(111)\right]}\left(\overrightarrow{r}\right)-\sum \limits_{\mu =1}^N{\rho}^{atom}\left(\overrightarrow{r}-\overrightarrow{R_{\mu }}\right)$. As shown in Fig. 5, the covalent and ionic bonding between V adatom and its adjacent Cu atoms are both clearly visible, for both the 1/9 ML and 1 ML coverages. Specifically, the covalent bonding is relatively stronger for 1/9 ML coverage (when compared with 1 ML), while the ionic bonding is relatively stronger for 1 ML coverage.

### Graphene Layer on Cu(111) Surface

The subT site is found to be the most stable adsorption site for V atoms on the clean Cu(111) from the adsorption energy in the above discussions. Although such an adsorption site is of some interests; however, V atoms staying underneath the Cu surface layer might lead to undesired property that limits its application as the catalyst at surface. Therefore, to alleviate the direct interaction between V adatoms and the Cu(111) surface, we try to introduce a buffer layer. Graphene is a perfect choice due to the most proximal lattice match with V/Cu(111) system, which has been firstly considered in this work.

**Fig. 6:**
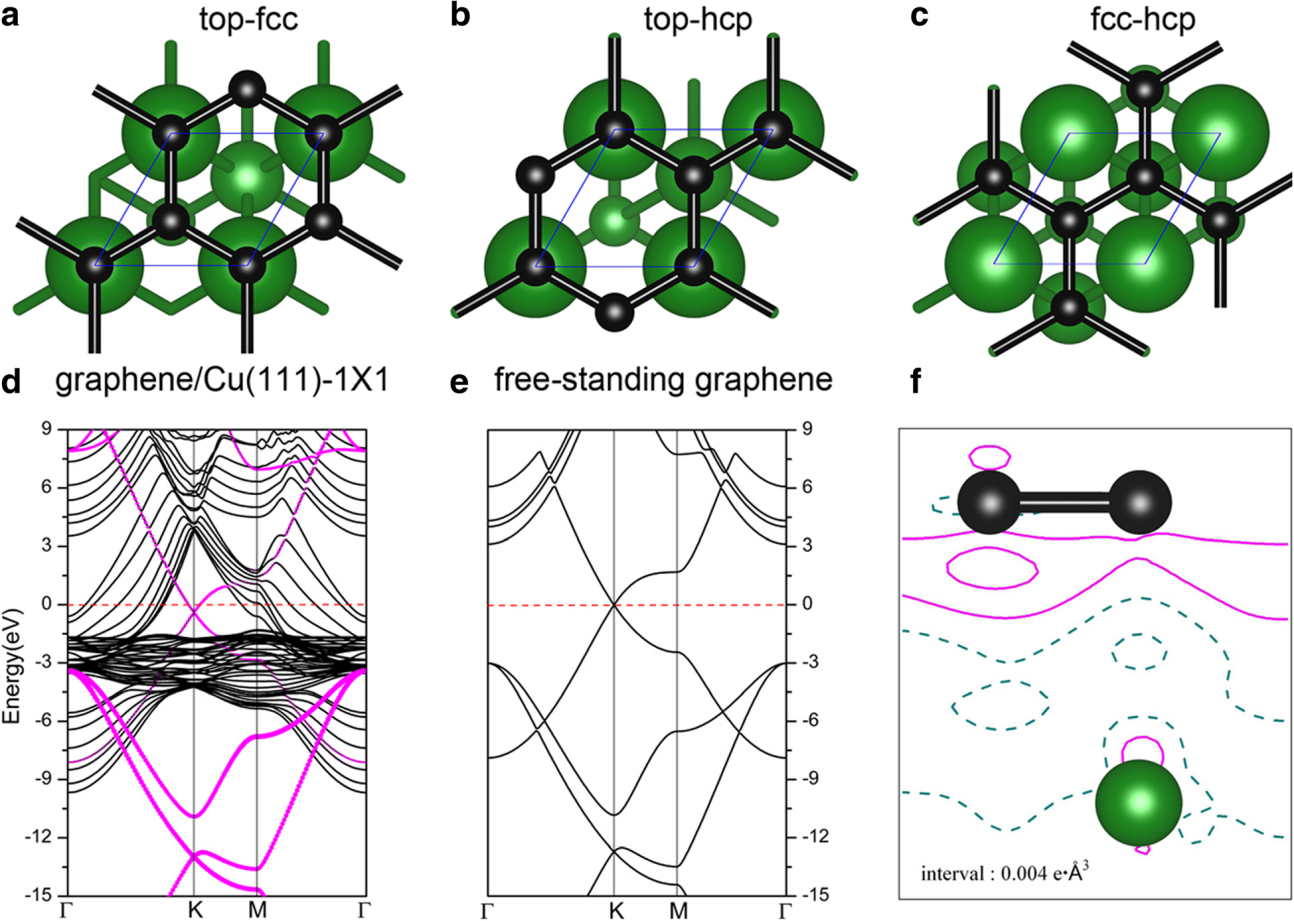
Geometry of graphene sheet on the Cu(111) surface: **a** top-fcc, **b** top-hcp, and **c** fcc-hcp sites. **d** Band structures of graphene/Cu(111) system in the top-fcc geometry, compared with **e** those of free-standing graphene. The magenta points indicate the electronic states contributed from graphene. **f** The charge differences between total charge density of graphene/Cu(111) and the sum of the charge densities of the independent graphene and the clean Cu(111) surface for the top-fcc geometry. The interval of the contour line is 0.004e Å^−3^

**Fig. 7:**
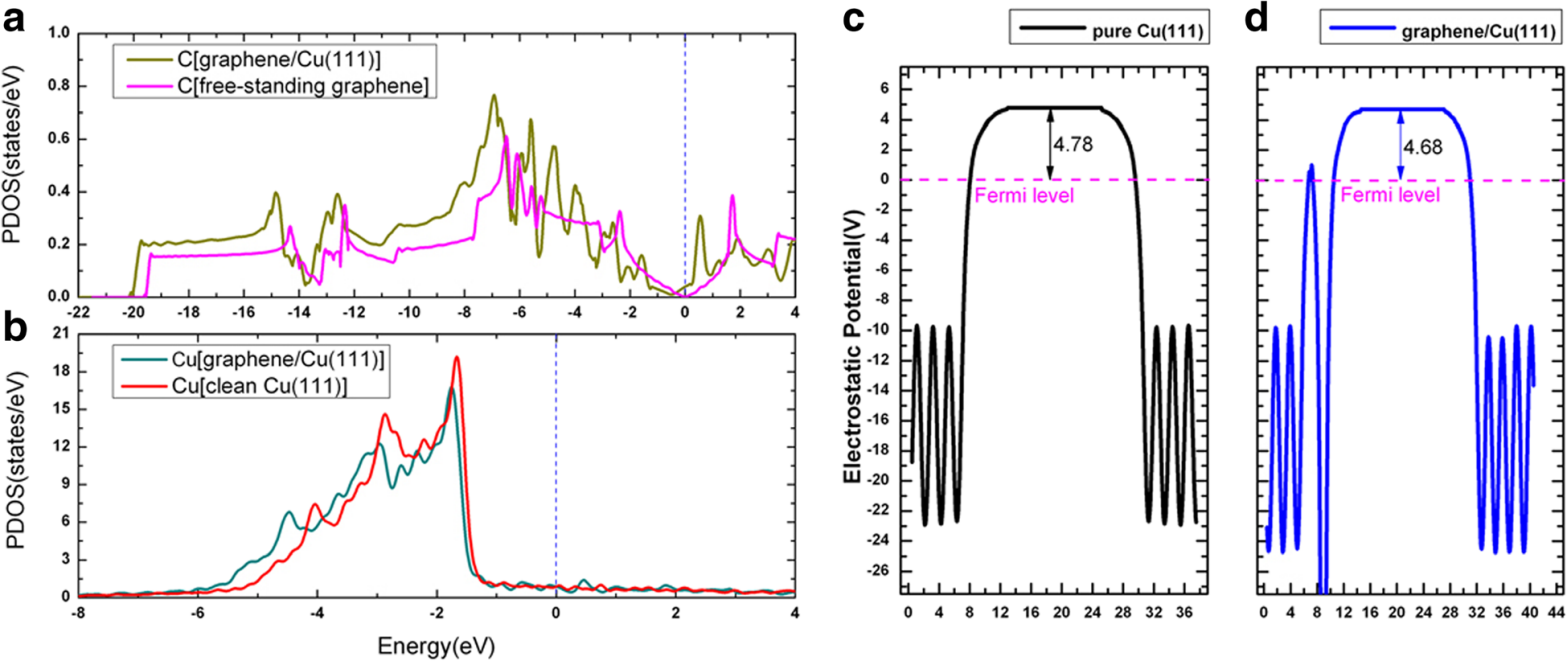
Projected density of states of graphene/Cu(111) for **a** C atoms and **b** Cu atoms. **c, d** Work function of a clean Cu(111) surface and a graphene/Cu(111) interface, respectively

Adsorptions of graphene on metal surfaces have been intensively studied in several previous publications [52–54]. When graphene is adsorbed on Cu(111), three possible geometries of the graphene/Cu(111) systems (aliased as Gra/Cu(111) hereafter) were considered, i.e., the graphene was on the top-fcc, top-hcp, and fcc-hcp adsorption sites; see Fig. 6a–c. Based on our results, the top-fcc geometry (Fig. 6a) is shown to be the energetically most stable structure, with an adsorption energy of 47 meV per carbon atom, and the equilibrium distance between graphene and Cu(111) surface is 3.14 Å, which agrees fairly well with the previous studies [52–54]. Such low adsorption energy (47 meV/C) and large interlayer distance imply that the binding between graphene and Cu(111) is relatively weak. Figure 6d shows the band structures of the graphene/Cu(111) system with the top-fcc configuration (Fig. 6a), especially, the electronic states contributed from graphene are sketched by magenta circles in the figure. The band structures of a free-standing graphene sheet are also shown in Fig. 6e for comparison. As can be seen from these figures, the band structures of graphene are very similar between the independent sheet and the one on the Cu(111) surface. The Dirac point at K (with linear band crossing) is preserved in Fig. 6d but with a little downshift when graphene is adsorbed on the Cu(111) surface. The downshift of the crossing point indicates a charge transfer from the Cu(111) substrate to the graphene layer, which is in accordance with the previous results that Al, Ag, and Cu are n-type doped by graphene [52–54]. The deformation charge densities, i.e., the charge differences between total charge density of Gra/Cu(111) and the sum of the charge densities of the independent graphene and the clean Cu(111) surface, i.e., $\Delta \rho \left(\overrightarrow{r}\right)={\rho}_{Gra/ Cu(111)}\left(\overrightarrow{r}\right)-{\rho}_{Gra}\left(\overrightarrow{r}\right)-{\rho}_{Cu(111)}\left(\overrightarrow{r}\right)$, are plotted in Fig. 6f. As shown in Fig. 6f, we can also observe the charge transfer from Cu(111) substrate to the graphene layer according to the solid contour lines around the C atoms. Meanwhile, we plot the projected density of states (PDOS) for C and Cu atoms in the Gra/Cu(111) system, together with the work function change of the Cu(111) surface (with and without graphene adsorption) in Fig. 7. From the integral of the PDOS, we found that the electrons on C atoms are slightly increased, while the electrons are slightly decreased for Cu atoms, which also verified the charge transfer phenomenon. Furthermore, we found that the calculated work function of Cu(111) changes from 4.78 to 4.68 eV after the adsorption of graphene layer. All of these confirm that charge transfer is from Cu substrate to the graphene layer.

### Vanadium Adsorption on Graphene-Covered Cu(111) Surface

In this section, we try to evaluate the atomic, electronic, and magnetic properties of vanadium adsorption on the graphene-covered Cu(111) surface. Four possible adsorption sites are considered, labeled as topA, bridge, topB, and hollow sites, as shown in Fig. 1d. The adsorption energies of V atoms on the Gra/Cu(111) at 1/9 ML and 1 ML are given in Fig. 1e, f, respectively. The energetically favored site for V adsorption on the graphene-covered Cu(111) surface is coverage-dependent. To be more specific, V adatoms prefer the maximally coordinated hollow sites (see Fig. 1d) for 1/9 ML, while the low coordinated top sites (i.e., topA site, see Fig. 1d) are preferred for high coverage of 1 ML. The adsorption energies, the bond lengths between V atom and its adjacent C atoms, and the atomic magnetic moments of vanadium and carbon for the V/Gra/Cu(111) systems are listed in Table 1. The adsorption energies of V atoms on the Gra/Cu(111) surface, E_ad_, are 1.91 and 1.16 eV per V atom for the 1/9 ML and 1 ML, respectively, which are reduced to some extent when compared with those on the Cu(111) surface. Obviously, the introduction of the graphene buffer layer can weaken the interaction between V adatoms and the Cu(111) surface as we expected. We further investigate the spin polarization energy of the ferromagnetic order of V in the V/Gra/Cu(111) at different coverages. The spin polarization energy is 390 meV for 1/9 ML, while there is no spin polarization for the 1 ML. The spin polarization energy is considerably higher in the V/Gra/Cu(111) system when compared with that in the V/Cu(111) system (390 meV compared with 110 meV, see Table 1). The magnetic moment of V adatom in the V/Gra/Cu(111) system at 1/9 ML is 2.93 μ_B_ which is close to 3 μ_B_/atom (the value of a gas-phase V atom), implying that V atoms are well isolated and there is little charge transferred between the V atom and the graphene layer. A small magnetic moment for C atom (0.16 μ_B_/atom) is also found.

**Fig. 8:**
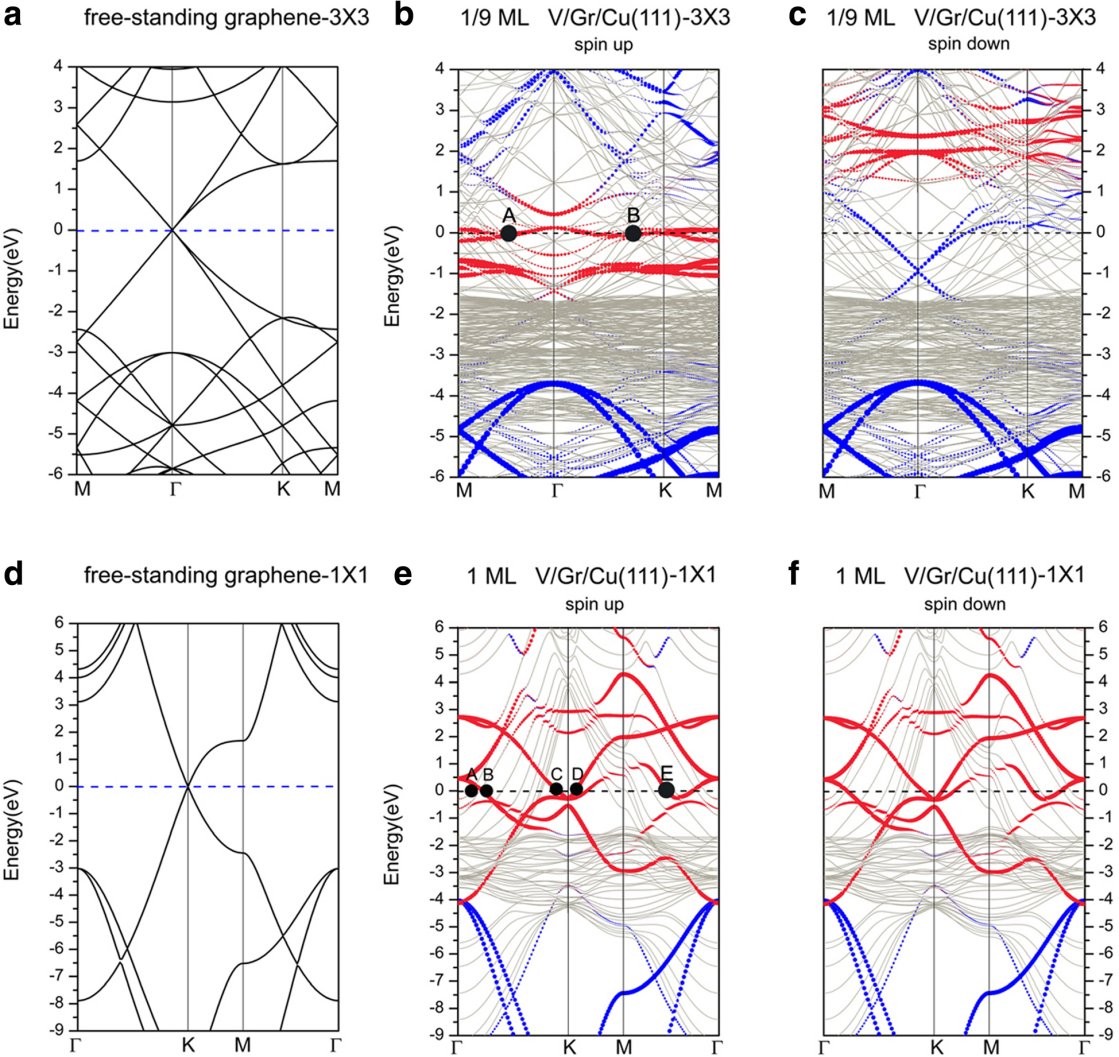
Band structures of a free-standing graphene, plotted in the Brillouin zones of **a** a (3 × 3) unit cell and **d** a (1 × 1) unit cell. Band structures of V adsorptions on the graphene-covered Cu(111) surfaces for **b** spin up of 1/9 ML, **c** spin down of 1/9 ML, **e** spin up of 1 ML, and **f** spin down of 1 ML

Next, we discuss on the band structures of V adsorption on the graphene-covered Cu(111) surface. Figure 8 depicts the band structures of the adsorption of V on the Gra/Cu(111) surface, together with the band structures of a free-standing graphene plotted in two different unit cells. Therein, the blue and red circles represent the contributions from the graphene and V adatoms, respectively. Firstly, it should be noted that there are many bands of Cu crossing the Fermi level, indicating that Cu atoms in the system contribute significantly to the conductance of the system. For 1/9 ML coverage, the band structures (see Fig. 8b, c) also show that the electronic structures of the system are spin-polarized. As mentioned in the previous section, the interaction between graphene and Cu(111) surface is very weak for Gra/Cu(111); accordingly, the bands contributed from graphene are easily recognized in the overall band structures. However, after V adsorption on the graphene-covered Cu(111) surface (i.e., V/Gra/Cu(111) system), we can see that the Dirac point of graphene is completely “destroyed” (see Fig. 8b) in the band structures of the spin-up channel, while the “linearly crossing point” of graphene is still distinguishable in the spin-down channel. Nevertheless, the very much downshift of “linearly crossing point” in the spin-down component indicates a relatively large number of charge transfer to the graphene layer. It should be noted that charges transferred to the graphene layer come from the layer of V atoms (see Fig. 10a), since the interactions between C and Cu layers are weak. For the spin-up channel at 1/9 ML, we found that except for a great deal of *d* electrons of substrate’s Cu atoms contribute to the Fermi surface, there are also lots of *d* electrons of V adatom contribution to the conductance of the system. Meanwhile, the hybridization of *p* electrons of C atoms with both *d* electrons of V adatom and surface Cu atoms is visible (but not remarkable). Likewise, we labeled two representative points (A, B) on the Fermi surface in Fig. 8b. Point A represents the contribution from the *d*_*xy*_, *d*_*x*_^*2*^ _*− y*_^*2*^ electrons of V adatom, while point B shows the contribution from the hybridization of *p*_*z*_ electrons of C atoms with both the *d*_*z*_^*2*^ electrons of V adatom and upmost layer of Cu atoms. Clearly, the surface V layer is an important conducting layer. In contrast, in the spin-down channel at 1/9 ML, the electronic states at the Fermi level mainly originate from *d* electrons of Cu atoms and *p*_*z*_ electrons of C atoms; the contribution of *d* electrons of V adatom is negligible. For 1 ML coverage, the band structures, shown in Fig. 8e, f, imply that the system is non-spin-polarized. Since the Dirac point of the graphene layer is also “destroyed,” therefore, the interaction between V adatoms and graphene buffer layer should be strong. From the calculated electronic states of the system, we see that electrons contributed to the Fermi level are mainly from the *s*, *d* electrons of Cu and *d* electrons of V atoms, as well as the *p*-electrons of C atoms. In more details, k-points which are located on the Fermi surface, A, B, C, D, and E, are labeled in Fig. 8e. Point A indicates the hybridization of *d*_*yz*_ and *d*_*z*_^*2*^ of the first layer (upmost) Cu atoms. Points B, C, and D are the electronic states from the *d*-electrons of V adatom. More specifically, point B describes the electron states from *d*_*xy*_, *d*_*x*_^*2*^ _*− y*_^*2*^ electrons of V adatom. Moreover, point E shows the strong hybridization of *s*, *d*_*z*_^*2*^ electrons of V adatom with *p*_*z*_ electrons of C atoms, together with relatively weaker mixing of *p*_*z*_ electrons of C atoms with *d*_*z*_^*2*^ electrons of the upmost layer of Cu atoms.

**Fig. 9:**
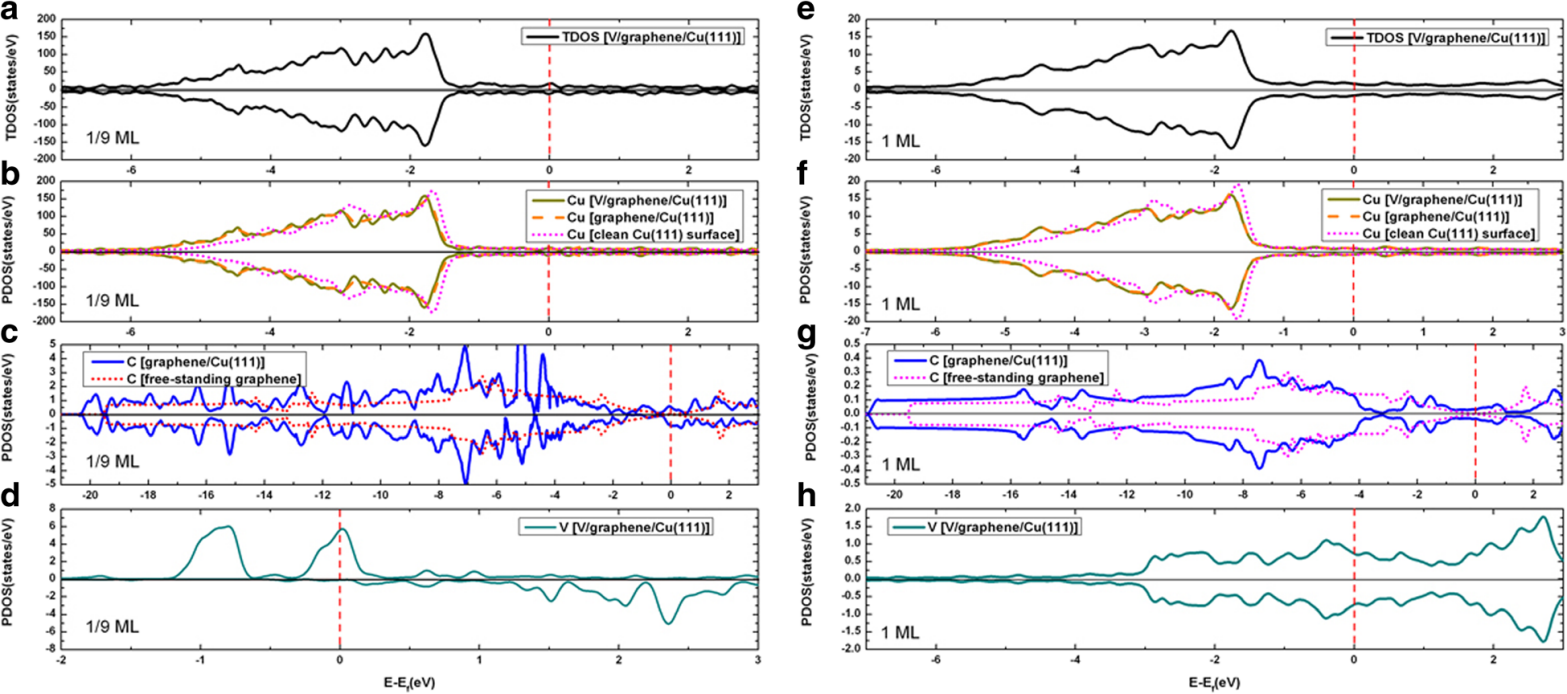
**a** TDOS of V adsorption on graphene-covered Cu(111) surface at 1/9 ML coverage; PDOS for **b** Cu atoms, **c** C atoms, and **d** V adatoms at 1/9 ML. Likewise, **e** TDOS of V adsorption on Gra/Cu(111) surface at 1 ML coverage; PDOS for **f** Cu atoms, **g** C atoms, and **h** V adatoms at 1 ML. For comparison, PDOS of Cu atoms of a clean Cu(111) and graphene/Cu(111) are implanted in **b** and **f**, while PDOS of C atoms of a free-standing graphene are also implanted in **c** and **g**

**Fig. 10:**
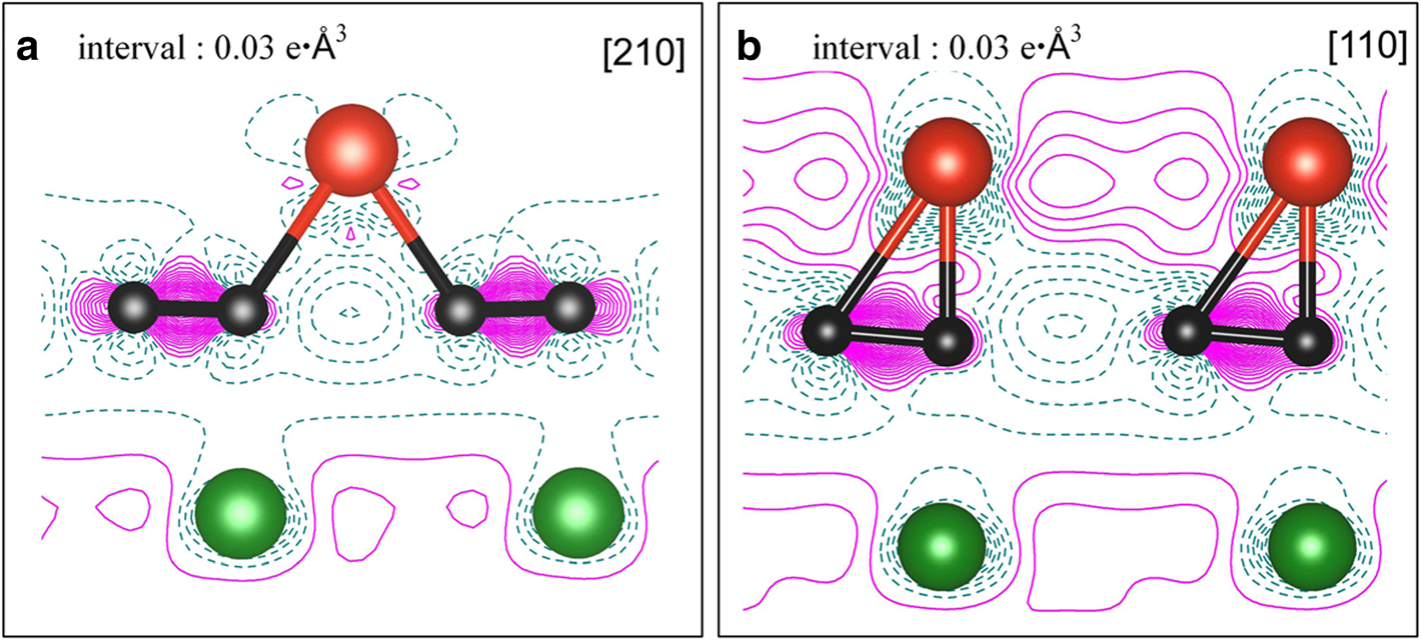
Deformation charge densities for the adsorption of V atoms on the graphene-covered Cu(111) surface at two coverages, i.e., **a** 1/9 ML and **b** 1 ML. Electron accumulation and depletion are represented by magenta solid lines and green dashed lines, respectively. The green, black, and red balls represent Cu, C, and V atoms, respectively

We now present the density of states for the V/Gra/Cu(111) system. The total density of states of V adsorption on the graphene-covered Cu(111) surface, together with the projected densities of states, are demonstrated in Fig. 9 for both the 1/9 ML and 1 ML coverages. At 1/9 ML, the spin polarizations of V adatoms and C atoms in the graphene layer are clearly seen (see Fig. 9c, d), while no spin polarization is found for Cu atom (see Fig. 9b). At 1 ML coverage, no spin polarization has been found for all atoms (see Fig. 9f-h). At 1/9 ML coverage, the DOS of the spin-up channel at the Fermi level is mainly contributed from the Cu atoms (totally 11.9 states/eV∙u.c.) and V atoms (totally 5.8 states/eV∙u.c.), with only minor contributions from the graphene layer (totally 0.4 states/eV∙u.c.). Meanwhile, the DOS of spin-down channel at the Fermi level is mainly contributed from the Cu atoms (totally 11.9 states/eV∙u.c.) and graphene layer (totally 1.1 states/eV∙u.c.), with only minor contributions from the V atoms (totally 0.1 states/eV∙u.c.). For the 1 ML coverage, both the DOS of spin-up and spin-down channels at the Fermi level are mainly contributed from the Cu atoms and V atoms (i.e., 1.1 and 0.7 states/eV∙u.c. for each spin component, respectively), with negligible contribution from the graphene layer (0.04 states/eV∙u.c). By integrating the PDOSs for each atom before and after the V adsorptions (leading to number of electrons), the charge transfer can be determined for different atoms. To be specific, the total valence electrons of the Cu atoms are reduced slightly for both the 1/9 ML and 1 ML coverages when compared with those of a clean Cu(111) surface, while the total valence electrons of C atoms are slightly increased when compared with that of a free-standing graphene. This implies that small amount of charges are transferred from Cu substrate to graphene layer for V/graphene/Cu(111) systems regardless of the V coverages. The total valence electrons of Cu atoms in V/Gra/Cu(111) systems are almost equal to those in the graphene/Cu(111) systems, which indicates that the Cu substrate has not been affected by V adsorption. The physical pictures given by the analysis of DOSs here are all in consistent with the analysis of the band structures. Finally, we show in Fig. 10 the contour plots of the deformation charge densities for the 1/9 ML and 1 ML coverages, respectively. The deformation charge density is defined as $\Delta \rho \left(\overrightarrow{r}\right)={\rho}_{\left[V/ Gra/ Cu(111)\right]}\left(\overrightarrow{r}\right)-\sum \limits_{\mu =1}^N{\rho}^{atom}\left(\overrightarrow{r}-\overrightarrow{R_{\mu }}\right)$. As shown in Fig. 10, the interactions between the graphene layers and the substrate Cu atoms are both relatively weak for 1/9 ML and 1 ML coverages, which are in consistent with the above discussions. From Fig. 10a, for the 1/9 ML, the bonding between V adatoms and its adjacent C atoms is mainly ionic, and the covalent bonding is not obvious. In contrast, for the 1 ML coverage, both ionic and covalent bonding between V adatom and its adjacent C atoms are clearly visible (see Fig. 10b). Besides, the covalent bonding between neighboring V adatoms is also very significant at 1 ML coverage. Due to the existence of graphene buffer layer, V adatoms cannot interact directly with the Cu atoms.

We have also calculated the phonon spectra for both the V/Cu(111) and V/Gra/Cu(111) systems. From the calculated phonon spectra, we find that there is no “imaginary frequency” for both the two types of systems, indicating that the systems studied are dynamically stable and would be seen in the laboratory. Since the main purpose of our work is not the thermodynamic stability, therefore the figures of the phonon dispersions are not shown in this text. Second, we have noticed that the different DFT functionals we adopted may lead to the different results. Hence, we have calculated the 1 ML V/Gra/Cu(111) system (as a representative) within the DFT framework under the B3LYP, HSE06 hybrid functionals, as well as the PBE functional. The results suggest that the adsorption site with largest adsorption energy is the topA site, calculated from all the PBE, HSE06, and B3LYP methods. However, relative adsorption energies at different adsorption sites from the B3LYP and PBE and HSE06 methods differ significantly (results from PBE and HSE06 methods are almost the same, since this is a metallic system). On the other hand, the geometrical parameters obtained from the three functionals show good consistency. Although the detailed charge density contours are somewhat different between PBE and B3LYP method, the main bonding characteristics are the same from both the two methods. In summary, the main point is that the adsorption energies obtained from B3LYP functional are significantly larger than those from the PBE and HSE06 functionals. To explain this point, Paier et al. argued that B3LYP functional lacked of a proper description of the “free-electron-like” systems with a significant itinerant character (e.g., metals and small gap semiconductors). They have concluded that the overestimation of the total energy of the atoms can be induced by the significantly overestimation on the exchange and correlation energies of B3LYP functional. In this respect, PBE functional often shows much more reliable results [55].

## Conclusions

To summarize, using first-principles calculations, we have systematically investigated the electronic and geometric properties of the adsorption of V atoms on both the clean Cu(111) surface and the graphene-covered Cu(111) surface. Firstly, for the V/Cu(111) system, an adsorption site underneath the Cu surface layer is found as the preferable adsorption site for V atom regardless of the coverages. The hybridization of V’s *d* states with Cu’s *d* states rules the electronic properties of V/Cu(111) systems. Ferromagnetic order of V adatoms is energetically favored for 1/9 ML coverage (1.34 μ_B_/atom), while no magnetism of V adatoms is observed for 1 ML coverage. Due to the strong interaction between V adatom and its adjacent substrate’s Cu atoms, the magnetic moment of V is significantly reduced. Secondly, the graphene/Cu(111) systems are investigated and the results agree well with the previous literatures. Thirdly, adsorptions of V on the graphene-covered Cu(111) at two coverages (i.e., 1/9 ML and 1 ML) show different preference of adsorption sites. The hollow site with maximum coordination is energetically favored for the adsorption of 1/9 ML, while the top site with low coordination is preferred for 1 ML adsorption. In V/Gra/Cu(111) systems, the interactions of C atoms with the V adatoms destroy the electronic properties of both the original graphene layer and the adsorbed atoms, represented by the strong hybridization of C’s *p*_*z*_-states with V adatoms’ *d*_*z*_^*2*^-states. A net magnetic moment for C atoms of graphene also appeared (0.16 μB/per carbon). In short, our study paves the way to a deep understanding of the adsorption properties of vanadium atoms on the clean Cu(111) and graphene-covered Cu(111) substrates. Simultaneously, this study also provides a reference for the possible applications of the V/Cu(111) and V/Gra/Cu(111) systems in the catalyst in nanomaterials industry, spintronic devices, and others.

## Abbreviations

Cu(111): (111) surface of copper
FM: Ferromagnetic
GGA: Generalized gradient approximation
ML: Monolayer
PAW: Projector augmented wave
PBE: Perdew-Burke-Ernzerhof
V/Cu(111): Vanadium atoms adsorbed on Cu(111) surface
V/Gra/Cu(111): Vanadium atoms adsorbed on graphene-covered Cu(111) surface
VASP: Vienna ab initio simulation package
vdWs: van der Waals interactions
